# Anthelmintic efficacy of mebendazole and levamisole in Olive Baboon (*Papio anubis*) infected with *Trichuris trichiura*

**DOI:** 10.1371/journal.pone.0326416

**Published:** 2025-07-17

**Authors:** Sohail Afzal, Uzma Farid Durrani, Asim Khalid Mahmood, Amber Fatima, Raheela Akhtar, Abdul Mateen, Amira S. H. Hassenin

**Affiliations:** 1 Department of Small Animal Clinical Sciences, University of Veterinary and Animal Sciences, Lahore, Pakistan; 2 Department of Pathology, University of Veterinary and Animal Sciences, Lahore, Pakistan; 3 Surgery Section, Department of Clinical Sciences, College of Veterinary and Animal Sciences, Jhang, Pakistan; 4 Faculty of Veterinary Medicine, Zagazig University, Zagazig, Egypt; Niigata University of Pharmacy and Medical and Life Sciences, JAPAN

## Abstract

The olive baboon (*Papio anubis*) belongs to the family Cercopithecoidea and lives in different environmental conditions as well as they are also kept in zoological gardens as a source of public entertainment. Poor growth and health disorders are commonly reported issues in olive baboons in captivity mainly because of gastrointestinal infections and endo-parasites. The present study was conducted on 15 dewormed olive baboons kept in Lahore Zoo, Lahore, Pakistan, and diagnosed with *Trichuris trichiura* (roundworm). Infected olive baboons were selected and randomly divided into 3 groups, i.e., A, B, and C. Group A and B were treated with mebendazole (20 mg/ kg, PO) and levamisole (10 mg/kg, PO), respectively. Group C was designated as a non-treated control group. Direct smear technique and simple fecal floatation technique were performed to diagnose positive samples. Pre- and post-treatment fecal screening was performed on days 0, 7, 14, 21, and 28. The effect of both anthelmintics on EPG values was calculated by pre- and post-treatment fecal screening. Egg per gram (EPG) was determined by using the McMaster technique. EPG values were used to determine the fecal egg count reduction (FECR). Statistically, there was a significant decrease in EPG values from days 0–14. On day 28 EPG values were significantly lower than day 0. There was a significant statistical difference between the treated and control groups P < 0.05, but there was no statistically significant difference between the mebendazole group and levamisole-treated groups (*P* > 0.05). Based on the numerical difference of mean EPG values and comparison between mebendazole and levamisole group; mebendazole was less effective than levamisole in olive baboons against *Trichuris* species. Repeated treatment was suggested for complete clearance of worm infestation in both treated groups.

## 1. Introduction

Olive baboons (*Papio anubis*) have existed for at least two million years. Baboons either live free in different geographic areas as wild primates or are kept in captivity in zoological gardens [1].

Parasitic worm infections are among the most neglected conditions within healthcare systems due to their chronic and often asymptomatic nature. Common symptoms include abdominal pain, diarrhea, malnutrition, gastrointestinal inflammation, an enlarged liver and spleen, pneumonitis, fatigue, eosinophilia, bowel obstruction, vomiting, constipation, blindness, lymphedema, anemia, weight loss, and itchy skin and anus. These symptoms highlight the detrimental effects of helminth infections on various body systems, underscoring the need for greater attention in healthcare interventions [2].

Different anthelmintics are used frequently in primates in Lahore Zoo, Lahore, Pakistan but the frequently faced challenges under tropical environment of the Lahore city of Pakistan to control worm infestation mainly include challenges of resistance & ongoing management [3].

The endoparasitic infection in baboons may also pose a zoonotic threat to humans residing in surrounding areas of wild animals. According to different studies, roundworm species *Oesophagostomum, Trichostrongylus* and *Trichuris* are the most common endoparasites of olive baboons [4]. In olive baboons, the prevalence of *Trichuris* and *Strongyloides* species are 26% and 60.7%, respectively [5].

For the identification of gastrointestinal parasites under field conditions, there are different fecal examination techniques including the most commonly employed direct smear technique, flotation, and sedimentation techniques. Direct smears have poor sensitivity because of the small number of feces examined but help to demonstrate the motile worms. Flotation and sedimentation techniques are the most common procedures. During the microscopic examination, the ova of the *Trichuris tichiura* appears barrel-shaped and brown. International CAPC recommends routine screening of feces by flotation technique [6].

*Trichuri*asis is a common intestinal whipworm of captive olive baboons [7]. All members of the genus *Trichuris* are nematode parasites that cause infection in the intestinal tract of a wide variety of mammals. *Trichuris* species are called whipworms because of their long filamentous bodies that thicken distally, resembling a bullwhip [8]. During infection, the anterior end of the adult worm is embedded in the intestinal mucosa while the posterior end is present in the lumen of the intestine. Among different gastrointestinal worms, whipworms are highly reported endoparasites [9].

Benzimidazole group of anthelmintics includes mebendazole, a broad-spectrum anthelmintic with high efficacy and a wide safety margin against *Trichuris trichiuria* (whipworm), *Enterobius vermicularis* (pinworm), *Ascaris lumbricoides* (roundworm), hookworm *Ancylostoma duodenale* and *Necator americanus*. This group is known to affect the tubulin protein of parasites [10]. The reason for the effectiveness of benzimidazoles lies in their ability to selectively bind to the tubulin protein within the parasites. This binding disrupts microtubule formation, which is essential for cellular functions like nutrient absorption and cell division. As a result, the worms are unable to maintain their cellular structure, leading to their eventual death. According to deworming strategies against gastrointestinal roundworms in olive baboons, mebendazole is also recommended [11]. According to different documented references, mebendazole can exhibit mild side effects, i.e., dizziness, diarrhea, pyrexia, and abdominal colic [12,13].

Levamisole and mebendazole are the most effective drugs against roundworms Strongyloides and Trichuris and other common nematodes [14]. Levamisole is a synthetic imidazothiazole derivative that has been widely used in treating worm infestations in both humans and animals. Levamisole is known for its efficacy against nematodes including *Ascaris, Trichostrongylus*, & various hookworms as well as its immune-boosting effects [15]. The selection of levamisole as a therapy option is based on its unique method of action, which includes activating the parasites nicotinic acetylcholine receptors at the neuromuscular junction. This stimulation produces persistent muscular contraction, resulting in paralysis and the worms evacuation from the host’s body. Levamisole’s capacity to quickly paralyze and remove nematodes, along with its potency at low dosages, makes it an important alternative in both human and veterinary medicine.

Considering the importance of an instantly effective anti*-Trichuris* anthelmintic in olive baboons in captivity, the present study was designed to evaluate the efficacy of 2 different anthelmintic drugs (mebendazole and levamisole) with an objective of significant reduction of EPG values in *Trichuris trichura* in captive olive baboons to improve their health as well as minimizing health risk not only to their neighboring animals but also to the visitors and caretakers.

## 2. Materials and methods

### Ethical approval

This study adhered to ethical criteria to protect the well-being of the animals involved. All protocols were evaluated and approved by the University of Veterinary and Animal Sciences (UVAS) Ethical Review Committee in Lahore, Pakistan, by Pakistani bylaws and OIE (World Organisation for Animal Health) standards for animal welfare in education and research. The study followed the highest ethical standards to minimize the animals’ misery and discomfort.

### Study area design and husbandry

The study was conducted on 15 non-dewormed olive baboons (*Papio anubis)* maintained and diagnosed with a heavy worm infestation. Baboons were maintained in semi-open enclosures with an open-air area (with sufficient natural light and air) for public exhibit, social and environmental interaction, and playing whereas the covered area was meant to ensure rest, peace during sleep time without any disturbance. Enclosures were also provided with natural vegetation and forage inside. Food was offered twice every day and comprised of bread, seasonal fruits, vegetables, nuts, and green leaves. Fresh water availability was always there in easy to access. Necessary measures were adopted for thermo regulation to combat the seasonal extremes. Food,water utensils,cage cleaning and disinfection were performed daily by the experienced keepers. To avoid boredom and keep the baboons physically active and alive; each cage was equipped with climbing items and other things, e.g., hanging ropes, tires, concrete shelves, log swings, and wall-mounted mirrors. The health status of baboons was determined by the zoo veterinarians once every 2–4 weeks or whenever needed mainly based on general health condition, body weight, appearance, general activity level, social response, and quantity of food intake. Prophylactic use of antibiotics (ciprofloxacin @ 10–20 mg/kg PO bid) for 3–5 days along with the multi-vitamins and other needed supplements was performed as per schedule especially in extreme weather conditions to avoid immune suppression linked infections, especially GIT and respiratory infections. Antibiotics were used to prevent immune suppression-related illnesses, particularly gastrointestinal (GI) and respiratory infections, which are more common under stressful settings. Extreme weather can damage an animal’s immune system, rendering them more vulnerable to opportunistic illnesses. The use of antibiotics in this context was intended to reduce the danger of such infections while also ensuring the animals’ overall health and well-being throughout the investigation.

### Sample collection

Sample collection and drug trials were performed in Lahore Zoo, Lahore, Pakistan inside baboon enclosures, and laboratory diagnostic work was conducted in the Diagnostic laboratory of the Department of Small Animal Clinical Sciences, University of Veterinary and Animal Sciences, Lahore, Pakistan. During this entire study, a total of 5 fecal samples per baboon (one sample at each stage) were collected in each group under this study for screening.

### Pre-study fecal screening

Fecal samples of 15 olive baboons*,* in this study, were collected and examined for the worms. **Colour:** Each fecal sample was visually scrutinized to document the color, which varied according to the baboons’ food and health status. The typical colors ranged from light to dark brown. Any aberrations, such as greenish, reddish, or blackish tints, were identified as possible markers of underlying health concerns or dietary impacts.

**Consistency:** The consistency of the fecal samples was noted, including whether they were formed, semi-formed, soft, or watery. Consistency is a key criterion for determining the gastrointestinal health of baboons. Formed stools are usually connected with a healthy digestive tract, but softer or watery stools may indicate diarrhea or mal-absorption disorders.

**Odor:** The odor of each sample was recorded, ranging from moderate to severe. An unusually bad odor may indicate an infection or a gut microbiota imbalance.

**Worms:** A comprehensive visual inspection was carried out to identify any apparent adult worm if present. Worms were collected and preserved in a preservative solution for later identification and analysis.

### Qualitative analysis of worm infestation

The microscopically observed ova and larvae were identified based on morphological features, i.e., size, color, and shape [16].

### Quantitative analysis for worm load

For quantitative analysis of worm burden egg per gram (EPG) count was determined using McMaster technique [17].

### Grouping of animals under study

Following the screening of fecal samples, 15 adult olive baboons of either sex were diagnosed as heavily infected with *Trichuris trichura* and randomly divided into 3 groups’ i.e., A, B, and C, each with 5 animals. *Trichuris trichura* is the only reported parasite in olive baboons in Lahore Zoo despite regular deworming and it appears to be resistant to various anthelmintics in this environment. Groups A and B were subjected to treatment trials with mebendazole (20 mg/Kg PO) and levamisole (10 mg/Kg PO), respectively. Group C was the non-treated control group.

### Pre and post-therapeutic fecal sampling

Fecal samples for the treatment trials were collected on days 07, 14, 21, and 28 and designated pre and post-treatment fecal samples. Pre and post-treatment fecal sampling was performed by collecting 10 go freshly passed feces of each baboon. Collected samples were sealed in plastic bags, labeled, and stored at 4°C till processing.

### Pre-treatment fecal screening

Before the administration of anthelmintic drugs, fecal samples; collected on day 0 in each group, were screened by macroscopic and microscopic examination. After examination, fecal egg count was performed by quantitative analysis of parasitic load to determine EPG.

### Treatment trials

The treatment trial was performed as a single administration of anti-helminthic drugs to evaluate its therapeutic efficacy and longevity of effects against *Trichuris* species. Group A and B were administered mebendazole (Syp.vermox®janssen100mg/5mL) and levamisole (Syp. Ketress ® ICI 40 mg/5mL), respectively [18]. Group C was the control group not receiving any treatment. Post-treatment protocols were as follows:

### Post-treatment fecal screening

Following administration of mebendazole and levamisole in groups A and B, fecal samples were collected and screened on days 7, 14, 21, and 28. Samples were screened by macroscopic and microscopic examination. A fecal egg count was performed to determine worm load and the anthelmintic efficacy was determined by fecal egg count reduction test (FECRT).


$$FECR\%\;=\;\frac{\left\{FEPGC\;(btm)\right\}-\{FEPGC\;(atm)\}}{\left\{FEPGC\;(btm)\right\}}\;\times 100\mathrm{\backslash ]}$$

FECR%= Fecal egg count reduction

FEPGC (BTM) =Fecal egg per gram count (Before treatment mean)

FEPGC (ATM) =Fecal egg per gram count (After treatment mean)

### Statistical analysis

The data obtained on fecal egg per gram (EPG) and fecal egg count reduction was statistically analyzed by repeated measures analysis of variance (ANOVA) with a significance level of 0.05% (P ≤ 0.05) using SPSS.

## 3. Results

Anthelmintic efficacy of mebendazole and levamisole were evaluated against *Trichuris trichiur*a in live baboons diagnosed positive for *Trichuris trichiur*a infestation. The results were based on the following parameters:

i **Identification and confirmation of gastrointestinal worm**

Microscopic examination of fecal samples revealed the presence of *Trichuris trichiur*a in all fecal samples of mebendazole, levamisole treated, and non-treated control baboons.

ii **Effect of Mebendazole and Levamisole on EPG and FECRP**

All mebendazole, levamisole treated and non-treated control group baboons were tested for fecal EPG and FEPGC qualitatively (Direct smear and flotation techniques) and quantitatively (McMaster technique and fecal egg count reduction test) on days 7, 14, 21, and 28. In mebendazole-treated baboons pre and post-treatment screenings showed a decrease in mean EPG values (Table 1) and an increase in FECRP from day 0–14 followed by a decrease on day 21 (S1 Fig). Statistical analysis revealed no significant difference among the mean EPG values on day 28 from days 7, 14, and 21. There was a significant difference between them in EPG values of days 0, 7, 14, 21, and 28 (P < 0.05). In levamisole-treated baboons, pre and post-treatment screening showed a decrease in mean EPG values (Table 1) and an increase in FECRP from day 0–14 followed by a decrease on day 21 (S1 Fig). On day 28 post treatment statistical analysis revealed a significant difference between them and EPG values on days 0, 21and 28 from days 7 and 14. There was no significant difference between days 14 and 21 (P > 0.05) but mean EPG values on days 0, 7, 14, 21, and 28 were significantly different (P < 0.05). In the non-treated control group, fecal screening showed an increase in mean EPG values (Table 1) and an increase in FECRP from days 0–28 (S1 Fig). Statistical analysis revealed mean EPG values statistically significant (P < 0.05) on days 0–28.

**Table 1:** Pre and post-treatment mean EPG values on days 0, 7, 14, 21, and 28.

Days	Mean *EPG ± S.E		
	Group A	Group B	Group C
0	710 ± 108.39	720 ± 135.09	710 ± 143.17
7	200 ± 79.05	320 ± 57.01	770 ± 75.82
14	30 ± 44.72	110 ± 65.19	810 ± 163.55
21	50 ± 61.23	120 ± 83.66	860 ± 155.72
28	80 ± 44.72	160 ± 65.19	900 ± 262.20

*EPG = Egg per Gram; S.E=Standard Error of Mean.

iii **Pre and post-treatment food intake (Kg)**

Under normal health conditions olive baboon were reported for consuming 2–2.5 kg food daily. At the beginning of this study food intake was recorded as very low to no food intake in all worms-infected baboons included in this study. Following the treatment with albendazole and levamisole, there was an improvement in food intake within 24 hours in each baboon. The increase in food intake was observed with an increase of mean food intake values from day 0–28. In non-treated baboon’s food intake remained low from day 0–28 (S2 Fig).

iv **Behavior**

Prior to the treatment a dull and depressed behavior was observed in all baboons involved in this study. Most of the time, baboons were seen sitting in one corner, showing the lowest activity level and response to any stimulus. Following the treatment with mebendazole and levamisole are markable improvement was observed in the general behavior and alertness of the treated baboons after and reduced worm burden? In non-treated baboons remained inactive and depressed throughout this study.

## 4. Discussion

The present study was conducted on adult olive baboons in captivity at Lahore Zoo, Pakistan. Baboons under this study were repeatedly diagnosed with *Trichuris trichura* as reported by [16,17,19], who also documented the frequent occurrence of *Trichuris trichura* in free-ranging baboons. According to different references *Trichuris* species prevalence is most common in all types and age groups of olive baboons. The transmission of *Trichuris* species is direct through the ingestion of first-stage infective larvae. Fecal shedding of *Trichuris* is highest in female lactating host sand less in the pregnant females. Adult *Trichuris* is found in the cecal region of the large intestine [20]. *Trichuris trichura* is also known for its potential to be transmitted to humans without causing a pathogenic effect like other zoonotic gastrointestinal parasites [16,20,21]. Levamisole and mebendazole are easily available in Pakistan and are effective as the anthelmintics against roundworms specially strongyloides and Trichuris [14,22]. Mebendazole is a clinically effective broad-spectrum anthelmintic drug from the benzimidazole group. The nematocidal effect of mebendazole takes place by inhibiting the synthesis of microtubules binding tone nematode tubulin, disrupting mitosis and mitochondrial functions that ultimately, especially of glucose leading to gradual immobilization and death of the helminthes [23,24]. According to earlier studies, mebendazole does not always cause side effects in animals under treatment at recommended dosages. Mild side effects of mebendazole may occasionally include dizziness, diarrhea, pyrexia, and abdominal alcohol [25,26]. The findings of the present study revealed that mebendazole-treated olive baboons did not show any side effects and were found to be safe [26]. Levamisole is a synthetic imidazothiazole derived anthelmintic, particularly roundworms, i.e., *ascaris, trichostrongylus*, and various hookworms especially *Ancylostoma* [15]. The mode of action of levamisole is gnostic proper ties against subtype nicotinic acetylcholine receptors in nematode body muscles. Due to agonistic activity, male worms lose control over their reproductive muscles and become unable to copulate. Levamisole is also associated with mild to severe side effects linked with the general health condition of the recipient and drug dosage. Besides the anthelmintics efficacy, levamisole is also known to boost the immune status of animals [27]. During this study use of levamisole in baboons was found to be quite safe and effective. No baboon elicited any side effects [14].

## 5. Conclusion

Based on the findings of this study it was concluded that statistically both mebendazole and levamisole cause a significant reduction of EPG values *in Trichuris trichura* in olive baboons. The numerical difference between the mean values *of Trichuris trichura* EPG values reduction by mebendazole and levamisole showed that the efficacy of mebendazole is better than that of the levamisole in olive baboons.

## Supporting information

S1 Fig Fecal egg count reduction percentage (FECRP). (TIFF)

S2 Fig Food intake in groups A, B and C. (TIFF)
