# Supplementary figures and images for: Anthelmintic efficacy of mebendazole and levamisole in Olive Baboon (Papio anubis) infected with Trichuris trichiura
Source: PLoS One. 2025 Jul 17;20(7):e0326416. doi: 10.1371/journal.pone.0326416 (PMC12270115; doi:10.1371/journal.pone.0326416)

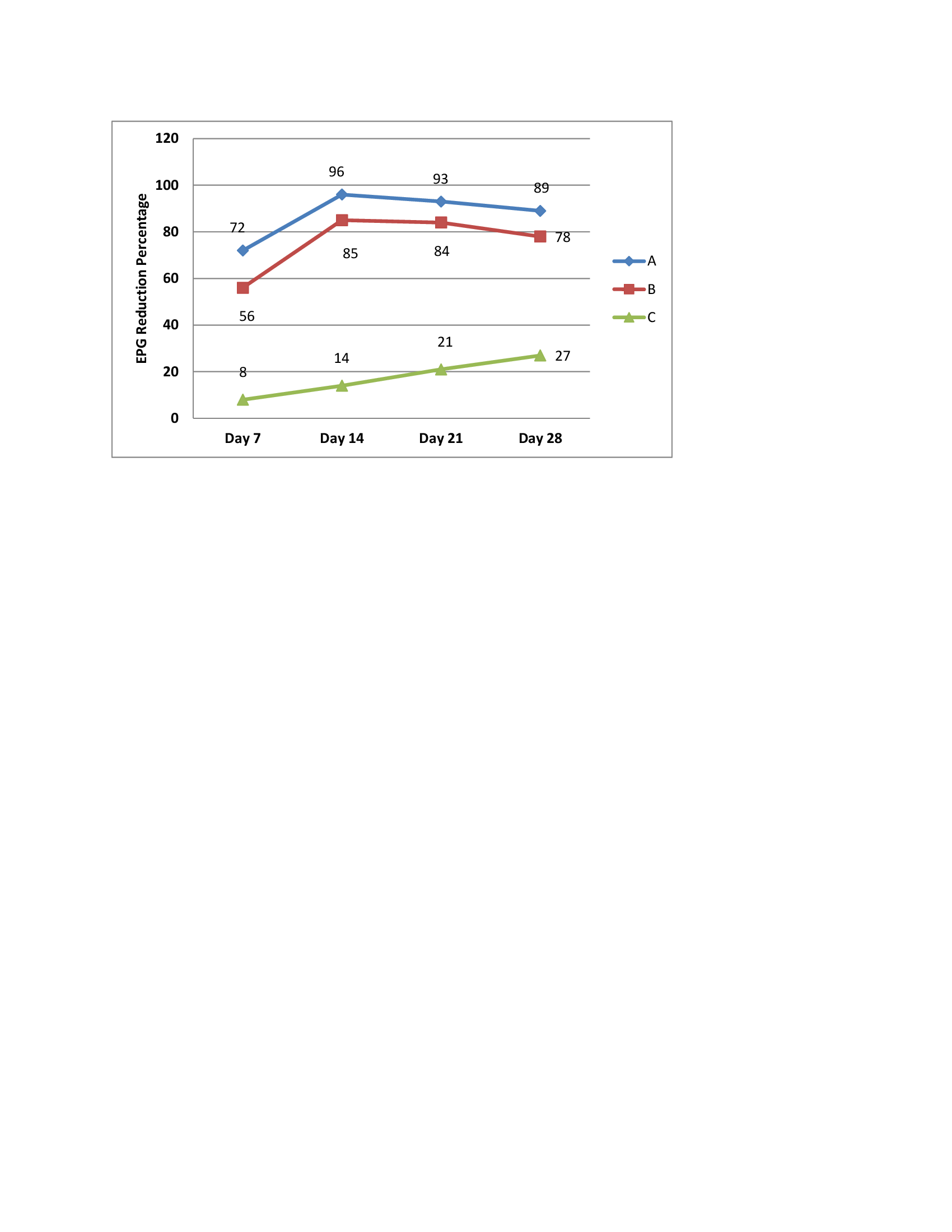

Supplement: S1 Fig — (TIFF) [file pone.0326416.s001.tiff]

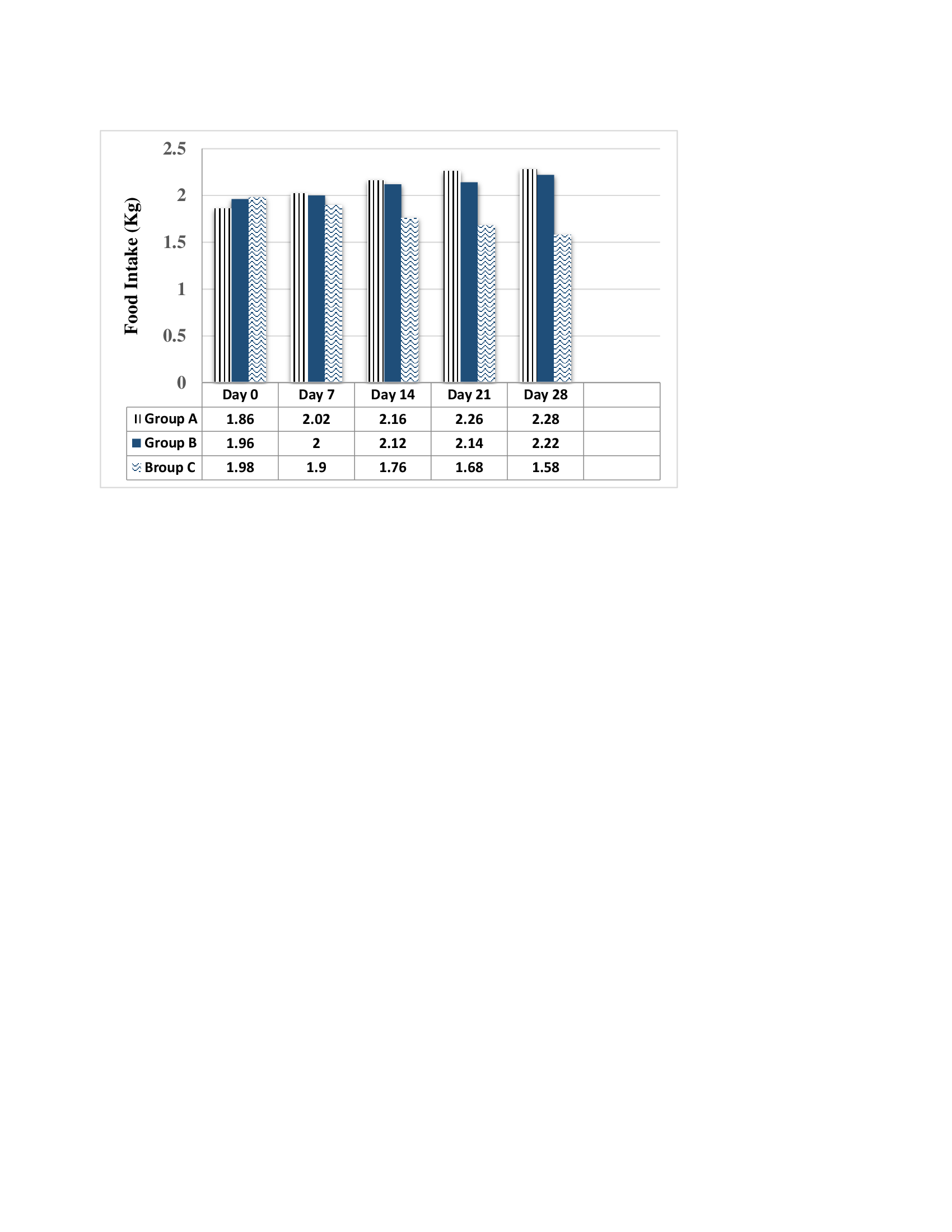

Supplement: S2 Fig — (TIFF) [file pone.0326416.s002.tiff]
